# (3-Carb­oxy-5-sulfonatobenzoato-κ^2^ *O* ^1^,*O* ^1′^)bis­[2-(2-pyrid­yl)-1*H*-benzimidazole-κ^2^ *N* ^2^,*N* ^3^]zinc(II) monohydrate

**DOI:** 10.1107/S1600536809009180

**Published:** 2009-03-19

**Authors:** Li-Juan Chen, Shen Lin, Ming-Xing Yang, Xiao-Yuan Wu

**Affiliations:** a College of Chemistry and Materials Science, Fujian Normal University, Fuzhou, Fujian 350007, People’s Republic of China; b State Key Laboratory of Structural Chemistry, Fujian Institute of Research on the Structure of Matter, Chinese Academy of Sciences, Fuzhou, Fujian 350002, People’s Republic of China

## Abstract

In the title compound, [Zn(C_8_H_4_O_7_S)(C_12_H_9_N_3_)_2_]·H_2_O, the Zn^II^ atom has a distorted octa­hedral coordination geometry, defined by four N atoms from two 2-(2-pyrid­yl)-1*H*-benzimidazole ligands and two O atoms from a deprotonated carboxyl­ate group of the 3-carb­oxy-5-sulfonatobenzoate ligand. In the crystal structure, the complex mol­ecules are linked into a three-dimensional network by inter­molecular O—H⋯O and N—H⋯O hydrogen bonds, and π–π stacking inter­actions with centroid–centroid separations of 3.758 (2) and 3.597 (1) Å.

## Related literature

For general background, see: Xia *et al.* (2005 ▶). For related structures, see: Kulynych & Shimizu (2002 ▶); Liu & Xu (2005 ▶); Sun *et al.* (2003 ▶); Xia *et al.* (2006 ▶).
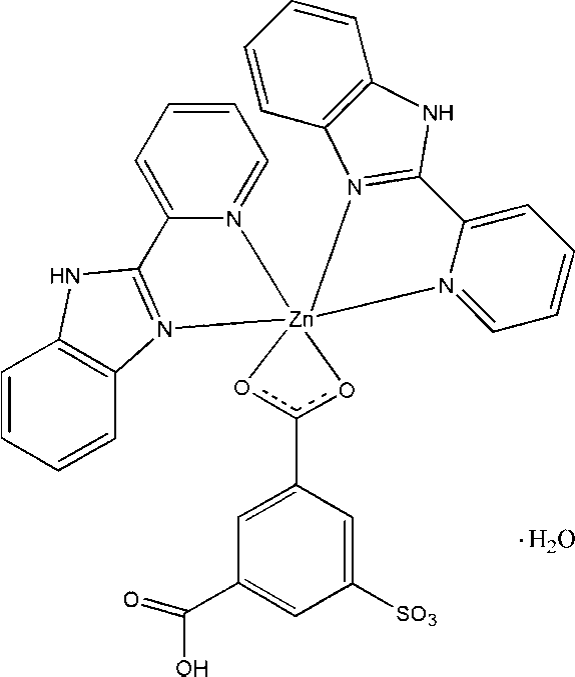


## Experimental

### 

#### Crystal data

[Zn(C_8_H_4_O_7_S)(C_12_H_9_N_3_)_2_]·H_2_O
*M* *_r_* = 718.00
Triclinic, 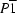

*a* = 11.086 (4) Å
*b* = 12.695 (5) Å
*c* = 13.347 (4) Å
α = 63.187 (10)°
β = 68.376 (13)°
γ = 87.122 (17)°
*V* = 1543.2 (10) Å^3^
*Z* = 2
Mo *K*α radiation
μ = 0.93 mm^−1^
*T* = 293 K
0.14 × 0.11 × 0.08 mm

#### Data collection

Rigaku Mercury CCD diffractometer
Absorption correction: multi-scan (*CrystalClear*; Rigaku, 2002 ▶) *T*_min_ = 0.843, *T*_max_ = 0.929
12166 measured reflections
6935 independent reflections
3857 reflections with *I* > 2σ(*I*)
*R*_int_ = 0.035

#### Refinement

*R*[*F* ^2^ > 2σ(*F* ^2^)] = 0.064
*wR*(*F* ^2^) = 0.179
*S* = 0.99
6935 reflections
434 parameters
H-atom parameters constrained
Δρ_max_ = 0.89 e Å^−3^
Δρ_min_ = −0.43 e Å^−3^

### 

Data collection: *CrystalClear* (Rigaku, 2002 ▶); cell refinement: *CrystalClear*; data reduction: *CrystalClear*; program(s) used to solve structure: *SHELXS97* (Sheldrick, 2008 ▶); program(s) used to refine structure: *SHELXL97* (Sheldrick, 2008 ▶); molecular graphics: *SHELXTL* (Sheldrick, 2008 ▶) and *DIAMOND* (Brandenburg, 1999 ▶); software used to prepare material for publication: *SHELXL97*.

## Supplementary Material

Crystal structure: contains datablocks global, I. DOI: 10.1107/S1600536809009180/hy2185sup1.cif

Structure factors: contains datablocks I. DOI: 10.1107/S1600536809009180/hy2185Isup2.hkl

Additional supplementary materials:  crystallographic information; 3D view; checkCIF report

## Figures and Tables

**Table 1:** Selected bond lengths (Å)

Zn1—N1	2.080 (4)
Zn1—N3	2.257 (4)
Zn1—N4	2.067 (4)
Zn1—N6	2.210 (4)
Zn1—O1	2.216 (3)
Zn1—O2	2.193 (3)

**Table 2:** Hydrogen-bond geometry (Å, °)

*D*—H⋯*A*	*D*—H	H⋯*A*	*D*⋯*A*	*D*—H⋯*A*
N2—H2*B*⋯O1^i^	0.86	2.15	2.884 (5)	143
N5—H5*B*⋯O3^ii^	0.86	2.05	2.833 (5)	151
O1*W*—H1*WA*⋯O7^iii^	0.82	1.87	2.678 (5)	167
O1*W*—H1*WB*⋯O6^iv^	0.82	2.05	2.825 (5)	157
O4—H4*B*⋯O1*W*	0.82	1.78	2.575 (4)	163

Symmetry codes: (i) 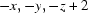; (ii) 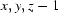; (iii) 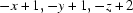; (iv) 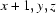.

**Fig. 1.**
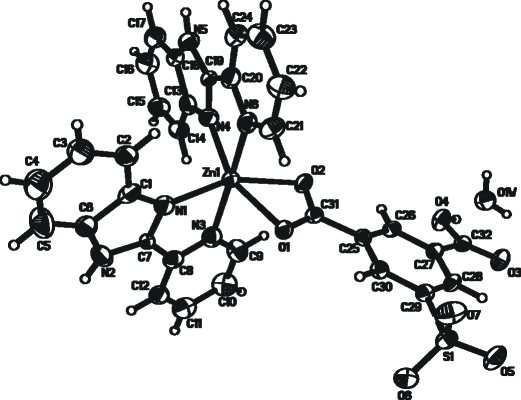
Molecular structure of the title compound. Displacement ellipsoids are drawn at the 30% probability level.

**Fig. 2.**
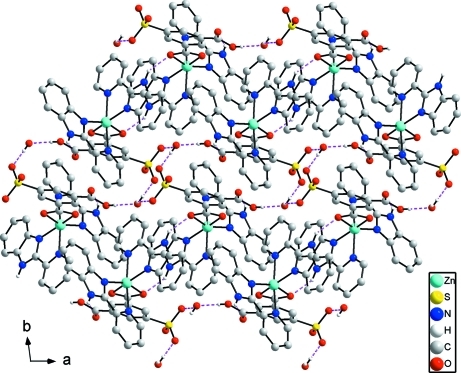
A perspective view of the crystal packing down the c axis, showing hydrogen bonds (dashed lines). H atoms, which do not participate in hydrogen bonds, have been omitted for clarity.

## supplementary crystallographic information

### Comment

Increasing interest has been focused on crystal engineering of supramolecular architectures organized by coordinating covalent bonds or supramolecular contacts such as hydrogen bonding and π–π interactions (Xia *et al.*, 2005). 5-Sulfoisophthalic acid (H_3_sipa), which exhibits variation in possible binding modes of the two carboxylate groups and the soft sulfonate group, and a strong tendency to form large, tightly bound metal cluster, has been demonstrated as a useful bridge ligand for the construction of supramolecular structures (Kulynych & Shimizu *et al.*, 2002; Liu & Xu *et al.*, 2005; Sun *et al.*, 2003). On the other hand, 2-(2-pyridyl)-1*H*-benzimidazole (2-pbim) ligand presents multiple N-donor sites with the possibility of reversible protonation and deprotonation, and has the capacity to act as a donor or acceptor in the formation of multi-dimensional hydrogen bonded networks (Xia *et al.*, 2006). We report here the crystal structure of the title compound, which contains both Hsipa and 2-pbim ligands.

As shown in Fig. 1, the title complex consists of one Zn^II^ atom, two neutral 2-pbim ligands, one deprotonated Hsipa^2-^ ligand and one uncoordinated water molecule. The Zn^II^ atom is six-coordinated by four N atoms from two 2-pbim ligands and two O atoms from one carboxylate group of the Hsipa^2-^ ligand, forming a distorted octahedral geometry (Table 1). The chelate rings A (Zn1, N1, C7, C8, N3), B (Zn1, N4, C19, C20, N6) and C (Zn1, O1, C31, O2) are oriented at dihedral angles of A/B = 84.1 (1)°, A/C = 87.8 (1)° and B/C = 82.4 (1)°. The two 2-pbim ligands bonded to the same Zn atom are nearly perpendicular to each other.

In the crystal structure, the mononuclear Zn complex molecules are linked by intermolecular O—H···O and N—H···O hydrogen bonds involving the water molecule, the imino groups, the carboxyl groups and the sulfonate group, forming a three-dimensional network (Fig. 2 and Table 2). The structure is further stabilized by π–π stacking interactions between the benzene rings of neighboring benzimidazole moieties containing N4 and N5 atoms, and between the pyridyl ring containing N3 atom and benzimidazole moiety containing N1 and N2 atoms, with centroid-to-centroid distances of 3.758 (2) and 3.597 (1) Å, respectively.

### Experimental

A mixture of Zn(NO_3_)_2_.6H_2_O (0.092 g, 0.3 mmol), NaH_2_sipa (0.053 g, 0.2 mmol), 2-pbim (0.039 g, 0.2 mmol) and H_2_O (10 ml) was placed in a 18 ml Teflon-lined Parr acid digestion bomb. The pH value of the reaction mixture was adjusted to *ca* 6.0 with 10% sodium hydroxide. The mixture was then heated for 3 d at 433 K under autogeneous pressure. Slow cooling of the reaction mixture to room temperature gave colorless prism crystals (yield: *ca* 78% based on Zn)

### Refinement

The water H atoms were located in a difference Fourier map and fixed in refinement with O—H = 0.82 Å and *U*_iso_(H) = 1.5*U*_eq_(O). Other H atoms were placed geometrically and refined as riding, with C—H = 0.93, O—H = 0.82 and N—H = 0.86 Å and with *U*_iso_(H) = 1.5*U*_eq_(O) or *U*_iso_(H) = 1.2*U*_eq_(C, N).

### Crystal data

**Table:**

[Zn(C_8_H_4_O_7_S)(C_12_H_9_N_3_)_2_]·H_2_O	*Z* = 2
*M**_r_* = 718.00	*F*(000) = 736
Triclinic, *P*1	*D*_x_ = 1.545 Mg m^−^^3^
Hall symbol: -P 1	Mo *K*α radiation, λ = 0.71073 Å
*a* = 11.086 (4) Å	Cell parameters from 3438 reflections
*b* = 12.695 (5) Å	θ = 2.4–27.5°
*c* = 13.347 (4) Å	µ = 0.93 mm^−^^1^
α = 63.187 (10)°	*T* = 293 K
β = 68.376 (13)°	Prism, colorless
γ = 87.122 (17)°	0.14 × 0.11 × 0.08 mm
*V* = 1543.2 (10) Å^3^	

### Data collection

**Table:**

Rigaku Mercury CCD diffractometer	6935 independent reflections
Radiation source: fine-focus sealed tube	3857 reflections with *I* > 2σ(*I*)
graphite	*R*_int_ = 0.035
Detector resolution: 14.6306 pixels mm^-1^	θ_max_ = 27.4°, θ_min_ = 2.4°
ω scans	*h* = −13→14
Absorption correction: multi-scan (*CrystalClear*; Rigaku, 2002)	*k* = −16→16
*T*_min_ = 0.843, *T*_max_ = 0.929	*l* = −14→17
12166 measured reflections	

### Refinement

**Table:**

Refinement on *F*^2^	Primary atom site location: structure-invariant direct methods
Least-squares matrix: full	Secondary atom site location: difference Fourier map
*R*[*F*^2^ > 2σ(*F*^2^)] = 0.064	Hydrogen site location: inferred from neighbouring sites
*wR*(*F*^2^) = 0.179	H-atom parameters constrained
*S* = 0.99	*w* = 1/[σ^2^(*F*_o_^2^) + (0.0855*P*)^2^] where *P* = (*F*_o_^2^ + 2*F*_c_^2^)/3
6935 reflections	(Δ/σ)_max_ < 0.001
434 parameters	Δρ_max_ = 0.89 e Å^−^^3^
0 restraints	Δρ_min_ = −0.43 e Å^−^^3^

### Fractional atomic coordinates and isotropic or equivalent isotropic displacement parameters (Å^2^)

**Table:**

	*x*	*y*	*z*	*U*_iso_*/*U*_eq_	
Zn1	0.29944 (5)	0.17840 (5)	0.78119 (5)	0.0450 (2)	
S1	0.04872 (11)	0.43789 (11)	1.21208 (10)	0.0462 (3)	
N1	0.1432 (4)	0.0898 (4)	0.7857 (3)	0.0502 (10)	
N2	0.0072 (4)	−0.0749 (4)	0.8695 (4)	0.0621 (11)	
H2B	−0.0332	−0.1461	0.9201	0.075*	
N3	0.2645 (4)	−0.0040 (4)	0.9412 (4)	0.0541 (10)	
N4	0.4499 (3)	0.1749 (3)	0.6348 (3)	0.0498 (10)	
N5	0.5526 (4)	0.2558 (3)	0.4336 (3)	0.0522 (10)	
H5B	0.5771	0.3064	0.3577	0.063*	
N6	0.3118 (4)	0.3556 (4)	0.6299 (4)	0.0507 (10)	
C1	0.0720 (5)	0.1113 (5)	0.7172 (4)	0.0524 (12)	
C2	0.0720 (5)	0.2091 (4)	0.6132 (4)	0.0537 (12)	
H2A	0.1273	0.2798	0.5780	0.064*	
C3	−0.0115 (6)	0.1981 (6)	0.5644 (6)	0.0768 (17)	
H3A	−0.0120	0.2631	0.4942	0.092*	
C4	−0.0948 (8)	0.0964 (7)	0.6132 (7)	0.107 (2)	
H4A	−0.1491	0.0938	0.5751	0.128*	
C5	−0.0999 (7)	−0.0010 (6)	0.7164 (6)	0.094 (2)	
H5A	−0.1585	−0.0693	0.7506	0.113*	
C6	−0.0172 (5)	0.0040 (5)	0.7683 (4)	0.0533 (12)	
C7	0.1051 (4)	−0.0216 (4)	0.8756 (4)	0.0337 (9)	
C8	0.1661 (4)	−0.0747 (4)	0.9639 (4)	0.0467 (11)	
C9	0.3292 (5)	−0.0428 (5)	1.0183 (5)	0.0565 (13)	
H9A	0.3984	0.0081	1.0031	0.068*	
C10	0.2964 (6)	−0.1531 (5)	1.1166 (5)	0.0701 (16)	
H10A	0.3428	−0.1773	1.1670	0.084*	
C11	0.1900 (6)	−0.2302 (5)	1.1404 (5)	0.0663 (15)	
H11A	0.1643	−0.3067	1.2057	0.080*	
C12	0.1258 (4)	−0.1844 (4)	1.0597 (4)	0.0489 (11)	
H12A	0.0539	−0.2308	1.0726	0.059*	
C13	0.5302 (4)	0.0978 (4)	0.6075 (5)	0.0496 (11)	
C14	0.5504 (5)	−0.0146 (4)	0.6844 (4)	0.0529 (12)	
H14A	0.5075	−0.0499	0.7687	0.063*	
C15	0.6384 (5)	−0.0690 (4)	0.6264 (5)	0.0598 (14)	
H15A	0.6554	−0.1435	0.6742	0.072*	
C16	0.7031 (5)	−0.0199 (5)	0.5016 (5)	0.0694 (16)	
H16A	0.7606	−0.0624	0.4684	0.083*	
C17	0.6843 (5)	0.0891 (5)	0.4266 (5)	0.0623 (14)	
H17A	0.7289	0.1229	0.3425	0.075*	
C18	0.5970 (4)	0.1477 (4)	0.4791 (4)	0.0408 (10)	
C19	0.4634 (4)	0.2702 (4)	0.5279 (3)	0.0365 (9)	
C20	0.3960 (4)	0.3701 (4)	0.5214 (4)	0.0494 (11)	
C21	0.2430 (4)	0.4475 (5)	0.6353 (5)	0.0545 (13)	
H21A	0.1845	0.4371	0.7110	0.065*	
C22	0.2562 (5)	0.5530 (5)	0.5352 (5)	0.0650 (15)	
H22A	0.2092	0.6138	0.5421	0.078*	
C23	0.3456 (5)	0.5667 (5)	0.4189 (5)	0.0713 (16)	
H23A	0.3580	0.6363	0.3475	0.086*	
C24	0.4123 (5)	0.4727 (4)	0.4171 (4)	0.0520 (12)	
H24A	0.4701	0.4792	0.3427	0.062*	
C25	0.3076 (4)	0.3139 (3)	0.9990 (4)	0.0342 (9)	
C26	0.4187 (4)	0.3155 (4)	1.0245 (4)	0.0365 (9)	
H26A	0.4935	0.2897	0.9866	0.044*	
C27	0.4184 (4)	0.3552 (3)	1.1057 (3)	0.0348 (9)	
C28	0.3057 (4)	0.3945 (4)	1.1619 (3)	0.0385 (10)	
H28A	0.3050	0.4219	1.2160	0.046*	
C29	0.1968 (4)	0.3927 (3)	1.1374 (4)	0.0374 (9)	
C30	0.1967 (4)	0.3535 (3)	1.0558 (4)	0.0373 (9)	
H30A	0.1223	0.3536	1.0391	0.045*	
C31	0.3042 (4)	0.2663 (4)	0.9162 (4)	0.0378 (9)	
C32	0.5345 (4)	0.3540 (4)	1.1378 (4)	0.0370 (9)	
O1	0.1971 (3)	0.2476 (3)	0.9116 (3)	0.0423 (7)	
O1W	0.8425 (3)	0.3154 (3)	1.1201 (3)	0.0623 (9)	
H1WA	0.8913	0.3655	1.0516	0.093*	
H1WB	0.8553	0.3320	1.1686	0.093*	
O2	0.4098 (3)	0.2450 (3)	0.8526 (3)	0.0477 (8)	
O3	0.5390 (3)	0.3947 (3)	1.2030 (3)	0.0506 (8)	
O4	0.6277 (3)	0.3028 (3)	1.0909 (3)	0.0538 (9)	
H4B	0.6863	0.2999	1.1152	0.081*	
O5	0.0764 (3)	0.4722 (3)	1.2907 (3)	0.0648 (10)	
O6	−0.0514 (3)	0.3350 (3)	1.2746 (3)	0.0621 (9)	
O7	0.0208 (3)	0.5357 (3)	1.1155 (3)	0.0687 (11)	

### Atomic displacement parameters (Å^2^)

**Table:**

	*U*^11^	*U*^22^	*U*^33^	*U*^12^	*U*^13^	*U*^23^
Zn1	0.0400 (3)	0.0546 (3)	0.0383 (3)	−0.0070 (2)	−0.0047 (2)	−0.0269 (3)
S1	0.0416 (6)	0.0549 (7)	0.0433 (6)	0.0136 (5)	−0.0115 (5)	−0.0285 (6)
N1	0.044 (2)	0.060 (2)	0.048 (2)	0.0038 (19)	−0.0146 (19)	−0.028 (2)
N2	0.055 (2)	0.059 (3)	0.062 (3)	−0.009 (2)	−0.020 (2)	−0.020 (2)
N3	0.045 (2)	0.064 (3)	0.062 (3)	0.011 (2)	−0.018 (2)	−0.039 (2)
N4	0.040 (2)	0.061 (2)	0.046 (2)	−0.0001 (19)	−0.0109 (18)	−0.027 (2)
N5	0.063 (2)	0.047 (2)	0.043 (2)	0.0088 (19)	−0.0158 (19)	−0.0215 (19)
N6	0.045 (2)	0.065 (3)	0.052 (2)	0.0038 (19)	−0.0195 (19)	−0.035 (2)
C1	0.047 (3)	0.077 (3)	0.041 (3)	0.015 (3)	−0.018 (2)	−0.034 (3)
C2	0.056 (3)	0.056 (3)	0.043 (3)	0.001 (2)	−0.023 (2)	−0.014 (2)
C3	0.075 (4)	0.083 (4)	0.066 (4)	−0.002 (3)	−0.038 (3)	−0.021 (3)
C4	0.118 (6)	0.119 (6)	0.085 (5)	−0.011 (5)	−0.061 (5)	−0.030 (5)
C5	0.104 (5)	0.084 (5)	0.099 (5)	−0.008 (4)	−0.064 (4)	−0.025 (4)
C6	0.056 (3)	0.059 (3)	0.043 (3)	0.004 (2)	−0.027 (2)	−0.015 (2)
C7	0.031 (2)	0.039 (2)	0.033 (2)	−0.0007 (17)	−0.0100 (17)	−0.0193 (19)
C8	0.043 (3)	0.050 (3)	0.046 (3)	0.014 (2)	−0.010 (2)	−0.028 (2)
C9	0.050 (3)	0.063 (3)	0.069 (3)	0.016 (2)	−0.040 (3)	−0.027 (3)
C10	0.068 (4)	0.071 (4)	0.072 (4)	0.027 (3)	−0.043 (3)	−0.023 (3)
C11	0.081 (4)	0.051 (3)	0.058 (3)	0.022 (3)	−0.023 (3)	−0.023 (3)
C12	0.039 (2)	0.059 (3)	0.051 (3)	0.009 (2)	−0.016 (2)	−0.029 (3)
C13	0.043 (3)	0.053 (3)	0.061 (3)	0.002 (2)	−0.019 (2)	−0.033 (3)
C14	0.062 (3)	0.041 (2)	0.046 (3)	0.005 (2)	−0.022 (2)	−0.011 (2)
C15	0.066 (3)	0.048 (3)	0.054 (3)	0.009 (3)	−0.022 (3)	−0.016 (3)
C16	0.067 (3)	0.065 (3)	0.075 (4)	0.019 (3)	−0.022 (3)	−0.036 (3)
C17	0.064 (3)	0.062 (3)	0.065 (3)	0.016 (3)	−0.017 (3)	−0.040 (3)
C18	0.042 (2)	0.044 (2)	0.040 (2)	0.007 (2)	−0.017 (2)	−0.022 (2)
C19	0.036 (2)	0.045 (2)	0.029 (2)	−0.0028 (18)	−0.0073 (17)	−0.0207 (19)
C20	0.048 (3)	0.058 (3)	0.051 (3)	0.007 (2)	−0.020 (2)	−0.031 (3)
C21	0.045 (3)	0.068 (3)	0.052 (3)	0.022 (2)	−0.015 (2)	−0.034 (3)
C22	0.049 (3)	0.068 (3)	0.069 (4)	0.011 (3)	−0.019 (3)	−0.027 (3)
C23	0.072 (4)	0.060 (3)	0.072 (4)	0.011 (3)	−0.032 (3)	−0.019 (3)
C24	0.050 (3)	0.064 (3)	0.051 (3)	0.015 (2)	−0.023 (2)	−0.032 (3)
C25	0.034 (2)	0.035 (2)	0.035 (2)	0.0031 (17)	−0.0150 (18)	−0.0161 (18)
C26	0.035 (2)	0.041 (2)	0.034 (2)	0.0098 (18)	−0.0120 (18)	−0.0193 (19)
C27	0.038 (2)	0.037 (2)	0.030 (2)	0.0065 (18)	−0.0163 (18)	−0.0146 (18)
C28	0.051 (3)	0.041 (2)	0.031 (2)	0.013 (2)	−0.0198 (19)	−0.0198 (19)
C29	0.039 (2)	0.037 (2)	0.036 (2)	0.0081 (18)	−0.0145 (19)	−0.0169 (19)
C30	0.033 (2)	0.039 (2)	0.042 (2)	0.0064 (18)	−0.0157 (19)	−0.0194 (19)
C31	0.043 (2)	0.036 (2)	0.031 (2)	0.0025 (19)	−0.0127 (19)	−0.0139 (18)
C32	0.039 (2)	0.042 (2)	0.030 (2)	0.0044 (19)	−0.0171 (19)	−0.0148 (19)
O1	0.0378 (16)	0.0528 (18)	0.0429 (17)	0.0016 (14)	−0.0164 (13)	−0.0265 (15)
O1W	0.0470 (19)	0.079 (2)	0.065 (2)	0.0038 (17)	−0.0263 (17)	−0.033 (2)
O2	0.0410 (17)	0.066 (2)	0.0487 (18)	0.0060 (15)	−0.0128 (14)	−0.0399 (17)
O3	0.061 (2)	0.063 (2)	0.0505 (19)	0.0203 (17)	−0.0378 (17)	−0.0336 (17)
O4	0.0399 (17)	0.081 (2)	0.069 (2)	0.0196 (17)	−0.0302 (17)	−0.052 (2)
O5	0.059 (2)	0.090 (3)	0.069 (2)	0.0218 (19)	−0.0220 (18)	−0.060 (2)
O6	0.0487 (19)	0.070 (2)	0.058 (2)	−0.0001 (17)	−0.0080 (16)	−0.0316 (19)
O7	0.064 (2)	0.073 (2)	0.055 (2)	0.0364 (19)	−0.0182 (18)	−0.0244 (19)

### Geometric parameters (Å, °)

**Table:**

Zn1—N1	2.080 (4)	C11—C12	1.401 (7)
Zn1—N3	2.257 (4)	C11—H11A	0.9300
Zn1—N4	2.067 (4)	C12—H12A	0.9300
Zn1—N6	2.210 (4)	C13—C14	1.398 (6)
Zn1—O1	2.216 (3)	C13—C18	1.421 (6)
Zn1—O2	2.193 (3)	C14—C15	1.373 (6)
S1—O5	1.431 (4)	C14—H14A	0.9300
S1—O7	1.453 (3)	C15—C16	1.381 (7)
S1—O6	1.453 (4)	C15—H15A	0.9300
S1—C29	1.798 (4)	C16—C17	1.355 (7)
N1—C7	1.332 (5)	C16—H16A	0.9300
N1—C1	1.348 (6)	C17—C18	1.371 (6)
N2—C7	1.353 (5)	C17—H17A	0.9300
N2—C6	1.387 (6)	C19—C20	1.423 (6)
N2—H2B	0.8600	C20—C24	1.368 (7)
N3—C8	1.303 (6)	C21—C22	1.364 (7)
N3—C9	1.366 (6)	C21—H21A	0.9300
N4—C19	1.351 (5)	C22—C23	1.437 (8)
N4—C13	1.371 (6)	C22—H22A	0.9300
N5—C19	1.367 (5)	C23—C24	1.376 (7)
N5—C18	1.377 (5)	C23—H23A	0.9300
N5—H5B	0.8600	C24—H24A	0.9300
N6—C20	1.335 (6)	C25—C30	1.390 (5)
N6—C21	1.377 (6)	C25—C26	1.397 (6)
C1—C2	1.384 (6)	C25—C31	1.490 (6)
C1—C6	1.449 (7)	C26—C27	1.384 (6)
C2—C3	1.356 (7)	C26—H26A	0.9300
C2—H2A	0.9300	C27—C28	1.399 (5)
C3—C4	1.364 (9)	C27—C32	1.497 (5)
C3—H3A	0.9300	C28—C29	1.367 (6)
C4—C5	1.357 (9)	C28—H28A	0.9300
C4—H4A	0.9300	C29—C30	1.386 (6)
C5—C6	1.355 (7)	C30—H30A	0.9300
C5—H5A	0.9300	C31—O1	1.251 (5)
C7—C8	1.458 (6)	C31—O2	1.267 (5)
C8—C12	1.345 (6)	C32—O3	1.211 (5)
C9—C10	1.362 (7)	C32—O4	1.306 (5)
C9—H9A	0.9300	O1W—H1WA	0.82
C10—C11	1.418 (8)	O1W—H1WB	0.82
C10—H10A	0.9300	O4—H4B	0.82
			
N4—Zn1—N1	100.37 (15)	C10—C11—H11A	122.0
N4—Zn1—O2	100.82 (13)	C8—C12—C11	121.3 (5)
N1—Zn1—O2	156.91 (13)	C8—C12—H12A	119.4
N4—Zn1—N6	77.61 (15)	C11—C12—H12A	119.4
N1—Zn1—N6	98.96 (15)	N4—C13—C14	130.1 (5)
O2—Zn1—N6	94.45 (13)	N4—C13—C18	109.4 (4)
N4—Zn1—O1	155.82 (14)	C14—C13—C18	120.5 (4)
N1—Zn1—O1	101.58 (13)	C15—C14—C13	115.1 (4)
O2—Zn1—O1	59.76 (10)	C15—C14—H14A	122.4
N6—Zn1—O1	89.04 (13)	C13—C14—H14A	122.4
N4—Zn1—N3	106.43 (14)	C14—C15—C16	124.1 (5)
N1—Zn1—N3	76.34 (16)	C14—C15—H15A	117.9
O2—Zn1—N3	88.94 (14)	C16—C15—H15A	117.9
N6—Zn1—N3	174.19 (13)	C17—C16—C15	121.0 (5)
O1—Zn1—N3	88.58 (13)	C17—C16—H16A	119.5
O5—S1—O7	113.4 (2)	C15—C16—H16A	119.5
O5—S1—O6	114.6 (2)	C16—C17—C18	117.6 (5)
O7—S1—O6	110.6 (2)	C16—C17—H17A	121.2
O5—S1—C29	106.1 (2)	C18—C17—H17A	121.2
O7—S1—C29	105.90 (19)	C17—C18—N5	133.7 (4)
O6—S1—C29	105.4 (2)	C17—C18—C13	121.7 (4)
C7—N1—C1	108.7 (4)	N5—C18—C13	104.6 (4)
C7—N1—Zn1	113.8 (3)	N4—C19—N5	109.7 (4)
C1—N1—Zn1	137.3 (4)	N4—C19—C20	122.6 (4)
C7—N2—C6	108.8 (4)	N5—C19—C20	127.7 (4)
C7—N2—H2B	125.6	N6—C20—C24	121.2 (4)
C6—N2—H2B	125.6	N6—C20—C19	113.3 (4)
C8—N3—C9	118.6 (4)	C24—C20—C19	125.5 (4)
C8—N3—Zn1	114.3 (3)	C22—C21—N6	123.3 (4)
C9—N3—Zn1	127.0 (4)	C22—C21—H21A	118.3
C19—N4—C13	107.0 (4)	N6—C21—H21A	118.3
C19—N4—Zn1	112.3 (3)	C21—C22—C23	117.5 (5)
C13—N4—Zn1	139.7 (3)	C21—C22—H22A	121.2
C19—N5—C18	109.3 (4)	C23—C22—H22A	121.2
C19—N5—H5B	125.3	C24—C23—C22	117.5 (5)
C18—N5—H5B	125.3	C24—C23—H23A	121.3
C20—N6—C21	118.5 (4)	C22—C23—H23A	121.3
C20—N6—Zn1	113.3 (3)	C20—C24—C23	121.9 (5)
C21—N6—Zn1	128.1 (3)	C20—C24—H24A	119.0
N1—C1—C2	133.3 (5)	C23—C24—H24A	119.0
N1—C1—C6	108.1 (4)	C30—C25—C26	119.0 (4)
C2—C1—C6	118.6 (4)	C30—C25—C31	119.7 (4)
C3—C2—C1	117.6 (5)	C26—C25—C31	121.3 (3)
C3—C2—H2A	121.2	C27—C26—C25	120.6 (4)
C1—C2—H2A	121.2	C27—C26—H26A	119.7
C2—C3—C4	123.3 (6)	C25—C26—H26A	119.7
C2—C3—H3A	118.4	C26—C27—C28	119.5 (4)
C4—C3—H3A	118.4	C26—C27—C32	121.7 (3)
C5—C4—C3	121.2 (6)	C28—C27—C32	118.8 (4)
C5—C4—H4A	119.4	C29—C28—C27	120.0 (4)
C3—C4—H4A	119.4	C29—C28—H28A	120.0
C6—C5—C4	118.3 (6)	C27—C28—H28A	120.0
C6—C5—H5A	120.9	C28—C29—C30	120.8 (4)
C4—C5—H5A	120.9	C28—C29—S1	121.1 (3)
C5—C6—N2	134.9 (5)	C30—C29—S1	118.2 (3)
C5—C6—C1	121.1 (5)	C29—C30—C25	120.2 (4)
N2—C6—C1	104.0 (4)	C29—C30—H30A	119.9
N1—C7—N2	110.4 (4)	C25—C30—H30A	119.9
N1—C7—C8	122.6 (4)	O1—C31—O2	121.4 (4)
N2—C7—C8	127.0 (4)	O1—C31—C25	119.3 (4)
N3—C8—C12	122.8 (5)	O2—C31—C25	119.3 (4)
N3—C8—C7	112.8 (4)	O1—C31—Zn1	61.3 (2)
C12—C8—C7	124.4 (5)	O2—C31—Zn1	60.2 (2)
C10—C9—N3	122.7 (5)	C25—C31—Zn1	178.0 (3)
C10—C9—H9A	118.7	O3—C32—O4	124.1 (4)
N3—C9—H9A	118.7	O3—C32—C27	122.6 (4)
C9—C10—C11	118.6 (5)	O4—C32—C27	113.3 (4)
C9—C10—H10A	120.7	C31—O1—Zn1	89.1 (2)
C11—C10—H10A	120.7	H1WA—O1W—H1WB	106.3
C12—C11—C10	116.1 (5)	C31—O2—Zn1	89.7 (3)
C12—C11—H11A	122.0	C32—O4—H4B	109.5

### Hydrogen-bond geometry (Å, °)

**Table:**

*D*—H···*A*	*D*—H	H···*A*	*D*···*A*	*D*—H···*A*
N2—H2B···O1^i^	0.86	2.15	2.884 (5)	143
N5—H5B···O3^ii^	0.86	2.05	2.833 (5)	151
O1W—H1WA···O7^iii^	0.82	1.87	2.678 (5)	167
O1W—H1WB···O6^iv^	0.82	2.05	2.825 (5)	157
O4—H4B···O1W	0.82	1.78	2.575 (4)	163

Symmetry codes: (i) −*x*, −*y*, −*z*+2; (ii) *x*, *y*, *z*−1; (iii) −*x*+1, −*y*+1, −*z*+2; (iv) *x*+1, *y*, *z*.

## References

[bb1] Brandenburg, K. (1999). *DIAMOND* Crystal Impact GbR, Bonn, Germany.

[bb2] Kulynych, A. D. & Shimizu, G. K. H. (2002). *CrystEngComm*, **4**, 102–105.

[bb3] Liu, Q.-Y. & Xu, L. (2005). *Inorg. Chem. Commun.* **8**, 401–405.

[bb4] Rigaku (2002). *CrystalClear* Rigaku Corporation, Tokyo, Japan.

[bb5] Sheldrick, G. M. (2008). *Acta Cryst.* A**64**, 112–122. 10.1107/S0108767307043930 18156677

[bb6] Sun, D.-F., Cao, R., Sun, Y.-Q., Bi, W.-H., Yuan, D.-Q., Shi, Q. & Li, X. (2003). *Chem. Commun.* pp. 1528–1529.

[bb7] Xia, C.-K., Lu, C.-Z., Yuan, D.-Q., Zhang, Q.-Z., Wu, X.-Y., Xiang, S.-C., Zhang, J.-J. & Wu, D.-M. (2006). *CrystEngComm*, **8**, 281–291.

[bb8] Xia, C.-K., Lu, C.-Z., Zhang, Q.-Z., He, X., Zhang, J.-J. & Wu, D.-M. (2005). *Cryst. Growth Des.* **5**, 1569–1574.

